# 

**Published:** 1861-11

**Authors:** 


### BOOKS RECEIVED.

*The Principles and Practice of Obstetrics. By* GUNNING S. BEDFORD, A. M., M. D., *Prof. of Obstetrics, the Diseases of Women and Children, and Clinical Obstetrics, in the University of New York; author of "Clinical Lectures on the Diseases of Women and Children." Illustrated by four Colored Lithographic Plates and ninety-nine Wood Engravings. 8vo. pp.* 763. *New York:* SAMUEL S. & WILLIAM WOOD, 389 *Broadway*, 1861.

*Medical Jurisprudence. By* ALFRED SWAINE TAYLOR, M. D., F. R. S., *Fellow of the Royal College of Physicians; Hon. M. D. Univ. St. Andrews; Member of the Royal College of Surgeons; and Professor of Medical Jurisprudence and Chemistry in Guy's Hospital. Fifth American from the Seventh and Revised London Edition. Edited, with additions, by* EDWARD HARTSHORNE, M. D., *one of the Surgeons to the Pennsylvania Hospital. Philadelphia:* BLANCHARD & LEA, 1861.

### MEDICAL NOTICES.

The Lecture, Introductory to the Course of Medical Lectures in the University of Buffalo, will be given on Wednesday, November 6th, at 10.30 A. M., in the Amphitheatre of the Medical College, by Prof. J. R. Lothrop. Doors open to the Profession and to all friends of the Institution.

---

### BUFFALO MEDICAL ASSOCIATION.

The regular meetings of this Society, for medical improvement, are held the first Tuesday evening, of each month, at the rooms, No. 7 South Division street, commencing at 7.30 P. M.

